# Obesity and Acute Kidney Injury in Patients with ST-Elevation Myocardial Infarction

**DOI:** 10.3390/jcm12237311

**Published:** 2023-11-25

**Authors:** Vojko Kanic, David Suran, Gregor Kompara

**Affiliations:** Division of Internal Medicine, Department of Cardiology and Angiology, University Medical Center Maribor, Ljubljanska Ulica 5, 2000 Maribor, Sloveniagregor.kompara@gmail.com (G.K.)

**Keywords:** myocardial infarction, acute kidney injury, body mass index, percutaneous coronary intervention, outcome

## Abstract

Background: Data on the association between obesity and acute kidney injury (AKI) in patients with ST-elevation myocardial infarction (STEMI) are sparse and inconclusive. We aimed to evaluate the association between obesity and AKI and the outcome in these patients. Methods: A retrospective observational study of 3979 STEMI patients undergoing percutaneous coronary intervention (PCI) was performed at a single center. Patients with and without AKI were compared. Patients were also divided into three categories according to BMI, and these were compared. All-cause mortality was determined at 30 days and over a median period of 7.0 years. Results: The incidence of AKI was similar in all BMI categories. There was no association between BMI categories and AKI (*p* = 0.089). The Spearman correlation coefficient between BMI categories and AKI showed no correlation (*r* = −0.005; *p* = 0.75). More AKI patients died within 30 days and in the long term [137 (18.5%) and 283 (38.1%) patients in the AKI group died compared to 118 (3.6%) and 767 (23.1%) in the non-AKI group; *p* < 0.0001]. AKI was harmful in all BMI categories (*p* < 0.0001) and was associated with more than a 2.5-fold and a 1.5-fold multivariable-adjusted 30-day and long-term mortality risk, respectively (aOR 2.59; 95% CI 1.84–3.64; *p* < 0.0001, aHR 1.54; 95% CI 1.32–1.80; *p* < 0.0001). BMI categories were not associated with 30-day mortality (*p* = 0.26) but were associated with long-term mortality (*p* < 0.0001). Overweight and obese patients had an approximately 25% lower long-term multivariable-adjusted risk of death than normal-weight patients. In patients with AKI, BMI was only associated with long-term risk (*p* = 0.022). Obesity had an additional beneficial effect in these patients, and only patients with obesity, but not overweight patients, had a lower multivariable adjusted long-term mortality risk than normal-weight patients (aHR 062; 95% CI 0.446–0.88 *p* = 0.007). Conclusions: In patients who experienced AKI, obesity had an additional positive modifying effect. Our data suggest that the incidence of AKI in STEMI patients is not BMI-dependent.

## 1. Introduction

Acute kidney injury (AKI), irrespective of the cause, is known to be associated with a worse prognosis in patients with myocardial infarction (MI) [1,2,3] and ST-elevation myocardial infarction (STEMI) [4,5]. AKI is a complex, multifactorial phenomenon that is common after percutaneous coronary intervention (PCI). It usually occurs in 3.5–14% of patients following PCI but can reach 50% in selected subsets of patients, particularly in those undergoing emergency PCI, those with chronic kidney disease (CKD), those with diabetes, and the elderly [1,2,3,4,6,7]. In addition to contrast volume, hemodynamic impairment, nephrotoxic drugs, inflammation, atheroembolism, and neurohormonal activation play important roles in the pathogenesis of AKI in emergency PCI [1,2]. There is a lack of effective treatment strategies [3]. Patients with STEMI usually require a higher contrast volume for PCI [1,3,6]. Unfortunately, fluid administration as a preventive measure against AKI is rarely feasible because of hemodynamic impairment, one of the major pathogenetic mechanisms of AKI in STEMI [3,8]. In addition, they require aggressive antithrombotic therapy to prevent thrombotic complications during treatment and sometimes mechanical circulatory support, pacemaker insertion, other arterial access, and central venous cannulation. This makes these patients highly susceptible to bleeding, another known predictor of AKI [6,9,10]. For all these reasons, patients with STEMI are more prone to AKI [2,5,11]. Due to the large number of percutaneous coronary interventions (PCIs) in the modern treatment of STEMI, AKI has increased clinical and socioeconomic importance [3].

Obesity has become a serious problem, with nearly 40% of adults being overweight [12]. Obesity is a known risk factor for the development of medical complications such as diabetes, hypertension, cardiovascular disease, acute coronary syndrome, cancer, and lung disease and carries an increased risk of a worse prognosis [12,13,14,15,16]. However, in patients with established coronary artery disease, overweight and patients with obesity have a more favorable prognosis, which is referred to as the “obesity paradox” [12,15,17].

The body mass index (BMI) is used most often to define categories of body weight because it can be measured very easily [16]. However, BMI does not accurately estimate body compartments, and it cannot discriminate between an excess of body fat and an increase in fat-free mass, which may result in nutritional misclassification [12,18,19]. In addition, it cannot discriminate between visceral and subcutaneous fat [16,18]. Visceral body fat has proatherosclerotic and cardio-depressant properties and carries a higher risk compared to subcutaneous fat [16,19]. The simplest method of assessing abdominal fat is waist circumference measurement [16], but this is rarely performed in patients with STEMI because this is a medical emergency.

The data on the association between BMI and AKI are inconsistent, inconclusive, and sometimes contradictory, and there is still no consensus on the link between BMI and outcomes in patients who suffer from AKI [20]. Obese patients are more likely to have insulin resistance, hypertension, diabetes, hyperlipidemia, and cardiovascular diseases, all of which are known to contribute to kidney disease [21,22], and they may be at higher risk for AKI [23]. In addition, renal vein congestion, pulmonary hypertension, and hypoventilation may contribute to kidney disease in patients with obesity [21]. For some, obesity is a risk factor for CKD [22], and patients with CKD are more susceptible to AKI [20,23,24,25]. Therefore, it can be inferred that patients with obesity are more likely to develop AKI [20]. Increased BMI has been designated as a profound risk factor for AKI in critically ill patients [17,21,23,26], although others have found a U-shaped risk for developing AKI with regard to BMI [20], or even that BMI <18.5 kg/m2 was independently associated with AKI [27]. For some, obesity is a clear risk factor for AKI [27,28], but this is not universally accepted [21].

The results of previous studies related to BMI and the outcomes of different patients who suffered from AKI are variable. Some analyses have found that obesity has some protective effect on mortality in patients with AKI compared with normal or underweight in critically ill patients [17,26], although others have found the opposite [21]. Yet others have found a U-shaped relationship between BMI and mortality in elderly postoperative patients with AKI [29]. In addition, some have found an inverse relationship between BMI and mortality in patients with AKI [30] and also in patients with AKI from various causes who underwent continuous renal replacement therapy [22]. A meta-analysis of 22 studies has shown that only underweight patients with AKI had a significantly higher mortality risk compared to normal-weight patients with AKI [20]. It has also been observed that patients with obesity after transcatheter aortic valve implantation who suffered AKI had better survival [31]. The association between BMI and AKI and the outcome is currently unknown [29]. It remains unclear whether obesity is truly an independent risk factor for AKI and whether it contributes to mortality in these patients [27]. It is also not clear whether patients with obesity and their comorbidities suffer more AKI during treatment of ST-elevation MI. There are some data indicating that BMI could beneficially modify the outcome in patients with STEMI with AKI [32]. Therefore, we aimed to investigate the possible association between BMI and the risk of AKI and outcome in patients with STEMI who underwent PCI.

## 2. Materials and Methods

### 2.1. Population

**Figure 1:**
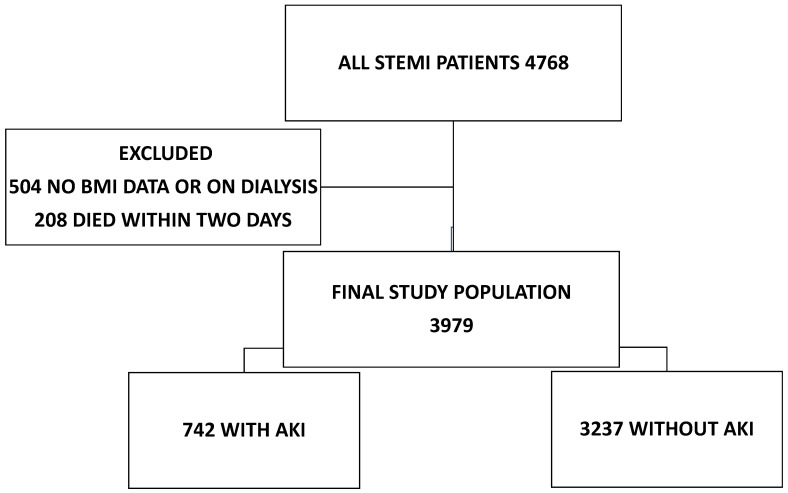
Patient selection. AKI = acute kidney injury; STEMI = ST-elevation myocardial infarction.

We performed a retrospective observational analysis of STEMI patients treated with PCI at the University Medical Centre Maribor between 1 January 2007 and 31 December 31 2018; the follow-up period ended on 30 July 2021. Data were collected from electronic medical records. A total of 4768 STEMI patients were treated with primary PCI during the study period, and their data were reviewed. Patients who were on dialysis or had no BMI (504 patients) and/or creatinine data (77 patients) were excluded. We also excluded patients who died in less than two days after admission (208 patients), because we considered that these patients would have had a fair chance of developing AKI if they had survived. This left 3979 patients for further analysis, 742 patients with and 3237 patients without AKI (Figure 1).

The study was approved by the institutional ethics committee (reference: UKC-MB-KME-59/19), and all methods were performed in accordance with relevant guidelines and regulations. Data included demographics, BMI, concomitant diseases (hypertension, diabetes, and hyperlipidemia), previous MI, CKD, and laboratory tests (creatinine, hemoglobin, troponin, and C-reactive protein on admission). Ventricular ejection fraction was determined by bedside echocardiography during the first 48 h after admission. In addition, clinical data included the presence of cardiogenic shock on admission, mechanical ventilation on admission, infused contrast volume, PCI of coronary vessels, multivessel PCI, access site, bleeding, and AKI.

### 2.2. Data Collection and Definitions

The diagnosis of STEMI was made in accordance with published guidelines, which were also followed in the management of patients [33,34]. The PCI strategy and concomitant medication were at the discretion of the operator and attending physician. The diagnosis of cardiogenic shock was made based on the accepted definition of a systolic blood pressure ≤90 mm Hg for ≥30 min or the need for supportive measures to maintain a systolic blood pressure of >90 mm Hg, clinical signs of pulmonary congestion, and signs of end-organ hypoperfusion. Thrombolysis in myocardial infarction (TIMI) flow grades were used to assess coronary blood flow [35]. Bleeding events were classified using the Bleeding Academic Research Consortium (BARC) definition, and BARC 3a bleeding was documented [36]. The patient’s body weight and height were determined using one of three methods: the weight was measured to the nearest 0.5 kg and height to the nearest 0.1 cm during the hospital stay, weight and height were self-reported, or the patient was weighed in a bed where the weight could be measured and the height was self-reported. The first serum creatinine level on admission was used to determine the initial renal function. The first blood sample was taken in the first 30 min after the first medical contact in the hospital and before PCI. Baseline renal dysfunction was defined as an estimated GFR less than 60 mL/kg/1.73 m^2^. GFR was calculated with the modification of diet in a renal disease study (MDRD) formula [37]. AKI was determined using the KDIGO criteria as an increase in serum creatinine of ≥0.3 mg/day within 48 h, or an increase in serum creatinine of at least 1.5 times the baseline that occurred within 7 days of admission [25]. Troponin levels were determined at admission and at least once in the first 24 h. After that, serum troponin levels were measured at various times at the discretion of the treating physician. Troponin I was determined by the chemiluminescence immunoassay method on Siemens Dimensions Vista Systems (Siemens Healthcare Diagnostics, Newark, DE, USA) with a reference interval of <0.045 µg/L. The highest serum troponin level during the entire hospitalization was defined as the peak troponin level. In previous studies, patients were classified differently with respect to BMI. We divided patients into three BMI categories: normal weight (<25.0 kg/m^2^), overweight (25.0–29.9 kg/m^2^), and obesity (BMI ≥ 30 kg/m^2^). All-cause mortality was assessed at 30 days and over a median period of 7.0 (25th, 75th percentile; 4,11) years to 30 July 2021. Mortality data were provided by the Slovenian National Cause of Death Registry.

### 2.3. End Points

The primary endpoint was the association between BMI and AKI in these patients. The secondary endpoint was the association between BMI and AKI and 30-day and long-term all-cause mortality. In addition, we assessed the possible impact of BMI outcomes in patients who suffered AKI.

### 2.4. Statistical Methods

The patients were divided according to the incidence of AKI to evaluate the contribution of BMI and other potential confounders. The Kolmogorov–Smirnov test was used to assess normal distribution. Differences between groups in baseline clinical, angiographic, and procedural characteristics were compared with the independent samples t-test, the Mann–Whitney U test or the Jonckheere–Terpstra test for continuous variables, and the chi-square test or Fischer’s exact test for categorical variables, as appropriate. A nonparametric Spearman’s Rho test was performed to identify correlations between BMI and AKI. Binary logistic regression models were performed using the enter mode to determine the possible association between BMI category and AKI and 30-day mortality, and Cox regression analysis was used to determine hazard ratios (HR) as estimates of long-term mortality. The models were adjusted for age, sex, hypertension, diabetes, hyperlipidemia, glomerular filtration rate, cardiogenic shock on admission, mechanical ventilation on admission, C-reactive protein on admission, access site, the volume of contrast infused, TIMI 0/1 after PCI, bleeding, troponin peak, P2Y12 receptor antagonists, BMI classes, and AKI. Only cardiogenic shock and ventilation on admission were used as variables. The variables included and retained in the model were based on previous literature reports and our experience that these factors are known to influence all-cause mortality. HRs were calculated with a stratified model according to the BMI value. All included variables had a univariable association with 30-day mortality *p* < 0.05 and a variance inflation factor (VIF) < 1.45. We calculated adjusted odds (OR) and hazard ratios (HR) for all groups. The normal weight category was used as the reference category in the models. Data were analyzed with SPSS 25.0 software for Windows (IBM Corp., Armonk, NY, USA). All *p*-values were two-sided, and values less than 0.05 were considered statistically significant.

## 3. Results

Of 3979 patients, 742 (18.6%) patients suffered AKI. These patients were older, more often had diabetes and CKD, but less often had hypertension and hyperlipidemia, more often suffered cardiogenic shock and were more frequently mechanically ventilated, and had lower GFR, higher serum creatinine, and higher C-reactive protein on admission. PCIs were more often performed with radial access, with a higher contrast volume, and they had more severe coronary artery disease (more PCIs for left main coronary artery disease) and more multivessel PCIs. In addition, they received P2Y12 less frequently but bled more often, had larger infarct sizes (higher troponin peaks), and lower ejection fractions. Otherwise, they were similar in terms of gender, BMI categories, and TIMI flow after PCI. Patient and procedural characteristics are shown in Table 1. Patient and procedural characteristics in different BMI categories are shown in Supplementary Table S1.

### 3.1. Association between BMI and AKI

The incidence of AKI was similar in all BMI categories (202 (27.2%), 314 (42.3%), and 226 (30.5%) patients in the normal weight, overweight, and obese categories, *p* = 0.13, respectively). There was no univariate association between BMI categories and AKI (*p* = 0.13). After adjusting BMI categories for confounding factors, they were not associated with AKI (*p* = 0.089) (Table 2). The Spearman’s correlation coefficient between the BMI categories and AKI showed no correlation (*r* = −0.005; *p* = 0.75). When we used BMI as a continuous variable, Spearman’s correlation coefficient between the BMI and AKI again showed no correlation (*r* = −0.006; *p* = 0.70).

### 3.2. AKI and Mortality in ST-Elevation MI Patients

**Figure 2:**
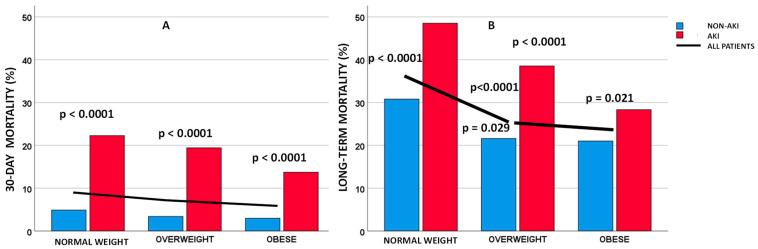
AKI and observed mortality in different BMI categories. (**A**) 30-day all-cause mortality; (**B**) Long-term all-cause mortality. AKI = acute kidney injury.

After 30 days, 137 (18.5%) patients with AKI died compared to 118 (3.6%) in the group without AKI; *p* < 0.0001 (Table 1). AKI was deleterious in all BMI categories [45 (22.3%) AKI patients vs. 39 (4.9%) non-AKI patients died in the normal weight category; *p* < 0.0001, 61 (19.4%) AKI patients vs. 51 (3.4%) non-AKI patients died in the overweight category; *p* < 0.0001, and 31 (13.7%) AKI patients vs. 28 (3.0%) non-AKI patients died in the obese category; *p* < 0.0001, respectively] (Figure 2A). The unadjusted AKI-to-non-AKI patient odds ratio for 30-day mortality was 5.98 (95% CI 4.61–7.77; *p* < 0.0001). After multivariable adjustment, AKI was associated with more than a 2.5-fold higher 30-day mortality (aOR 2.59; 95% CI 1.84–3.64; *p* < 0.0001). Associations of all variables in the model with 30-day and long-term mortality are shown in Table 3.

After the end of the observation period, 1050 (26.4%) patients had died. More patients with AKI had died 283 (38.1%) compared to 767 (23.1%) patients without AKI; *p* < 0.0001 (Table 1). In addition, AKI patients were more likely to die in the long-term in all BMI categories [98 (48.5%) AKI patients vs. 246 (30.8%) non-Aki patients died in the normal weight category; *p* < 0.0001, 121 (38.5%) AKI patients vs. 324 (21.6%) non-AKI patients died in the overweight category; *p* < 0.0001, and 64 (28.3%) AKI patients vs. 197 (21.0%) non-AKI patients died in the obese category; *p* = 0.021, respectively] (Figure 2B). The unadjusted AKI patient-to-non-AKI patient odds ratio for long-term mortality was 1.99 (95% CI 1.68–2.35; *p* < 0.0001). After multivariable adjustment, patients with AKI had more than a 50% higher risk of dying in the long term compared to patients without AKI (aHR 1.54; 95% CI 1.32–1.80; *p* < 0.0001). The associations of all variables with 30-day and long-term mortality are shown in Table 3.

### 3.3. BMI Categories and Mortality

**Figure 3:**
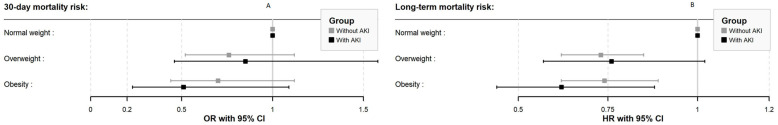
Association between BMI categories and 30-day and long-term mortality risk in STEMI patients with and without AKI. (**A**) 30-day mortality risk; (**B**) Long-term mortality risk. AKI = acute kidney injury; BMI = body mass index; STEMI = ST-elevation myocardial infarction.

The mortality after 30 days decreased inversely with BMI (84 (8.4%) patients died in the normal weight category, and 112 (6.2%) and 59 (5.1%) in the overweight and obese categories; *p* = 0.006, respectively (Figure 2A). A univariate association between BMI categories and 30-day mortality was observed (*p* = 0.007). However, after multivariable adjustments, there was no association between BMI categories and 30-day mortality (*p* = 0.26) (Table 3, Figure 3).

At the end of the observation period, we found an inverse relationship between mortality rate and BMI category. The mortality rate was highest in the normal weight category and significantly lower in the overweight and obese categories (344 (34.4%), 445 (24.5%), and 261 (22.4%); *p* < 0.0001, respectively (Figure 2B). A univariate association between BMI categories and long-term mortality (*p* < 0.0001) was observed, with overweight patients and patients with obesity having a lower risk than normal weight patients (HR 0.66; 95% CI 0.58–0–76; *p* < 0.0001, and HR 0.60; 95% CI 051–0.71; *p* < 0.0001, respectively). After multivariable adjustment, BMI categories were associated with long-term mortality (*p* < 0.0001). Overweight patients and patients with obesity had a 27% and 26% lower long-term multivariable adjusted mortality risk than normal weight patients (aHR 073; 95% CI 0.62–0.89; *p* < 0.0001, and aHR 074; 95% CI 0.62–0.89; *p* = 0.002, respectively) (Table 3, Figure 3). Overweight patients and patients with obesity had a similar risk when we compared only overweight patients and patients with obesity (*p* = 0.73).

### 3.4. Association between BMI Categories and Outcome in ST-Elevation MI Patients Who Suffered AKI

In STEMI patients with AKI, the observed all-cause 30-day mortality was similar in all BMI categories (*p* = 0.062) (Figure 2A). There was a univariate association between BMI and 30-day mortality (*p* < 0.0001), and overweight patients and patients with obesity had a lower risk of 30-day mortality (OR 0.66; 95% CI 0.46–0.95; *p* = 0.025, and OR 0.42; 95% CI 0.28–0.62; *p* < 0.0001). However, after adjusting for confounding factors, this association was not significant in 30-day mortality (*p* = 0.20) (Table 4, Figure 3A). At the end of the observation period, we observed an inverse relationship between the mortality rate and BMI category (Figure 2B). After adjusting for confounding factors, this association was significant in the long term (*p* = 0.022). Furthermore, only patients with obesity had more than a 35% lower multivariable adjusted long-term mortality risk compared to normal-weight patients (aHR 062; 95% CI 0.44–0.88; *p* = 0.007), while overweight patients had a similar risk of long-term death compared to normal-weight patients (aHR 0.76; 95% CI 0.57–1.02; *p* = 0.067) (Table 4, Figure 3).

## 4. Discussion

AKI is well known to be associated with the outcome of MI [4,5,12,21]. In addition, BMI is associated with the outcome of patients with MI [15,38]. Some data indicate that increased BMI could have a beneficial modifying effect in STEMI patients with AKI [32]. In our long-term follow-up study, we followed patients for up to 12 years. We found that obesity had an additional beneficial effect on patients with AKI. Only obese patients had a lower multivariable adjusted long-term mortality risk compared with normal-weight patients, whereas in patients without AKI, overweight patients also had a lower risk.

Our finding confirms the previous observation by Schwartz et al., who found a positive modifying effect of BMI in patients with STEMI who suffered from AKI [32]. In STEMI patients with AKI, obesity was a “protective” factor in terms of mortality risk. Apparently, age, hemodynamic impairment, bleeding, access site, and medications are more important for the development of AKI than a higher BMI (with all its shortcomings) in patients with STEMI. This would explain the similar incidence of AKI in all BMI categories and why contrast volume was not associated with AKI (there was only an eight-ml difference between groups). Patients with STEMI represent a population with a known, curable cause (coronary artery obstruction) and some modifiable treatment modalities (artery opening, bleeding prevention, hemodynamic stability, nephrotoxic drugs, contrast volume, and preventive hydration) that may not be present in other critically ill patients. This may explain the difference between our result and the results of other studies in critically ill patients of other etiologies in whom BMI predicted AKI [17,21,23,26]. Consistent with previous results, we found an association between BMI and outcome in patients with AKI in whom the lowest risk of death shifted to the right (toward obesity) [29]. In STEMI (and other acute events), activation of the neurohormonal system and inflammation suddenly require additional energy, which could be provided by extra metabolic reserves stored in subcutaneous fat [15]. Patients with AKI were older, had more comorbidities, were more likely to be in cardiogenic shock, had more inflammation (higher C-reactive protein), suffered more bleeding, and had a larger infarct size. Sudden metabolic demands were higher compared with non-AKI patients, making the lower risk of death in patients with obesity with more caloric reserves (compared with normal-weight patients) more understandable. These reserves were not sufficiently different (“higher”) in overweight patients (compared with normal-weight patients) to ensure a lower risk of death. In critically ill patients with AKI requiring renal replacement therapy, patients with obesity were also found to have the lowest mortality risk [17]. This supports the assumption of a higher need for metabolic reserve in these high-risk patients [15]. Another possible explanation could be the protective effect of obesity against inflammation, which is one of the most important pathophysiological problems in AKI [27]. Patients with obesity have higher lipoprotein levels, which protect the endothelium in renal vessels against endotoxins [27]. The patients with obesity in our analysis had higher cholesterol and triglyceride levels and lower HDL cholesterol levels at admission compared with normal-weight patients (*p* = 0.037, *p* < 0.0001, *p* < 0.0001, respectively) (Supplementary Table S1). A possible methodological explanation could be that patients with more concomitant diseases (malignancies, occult diseases, gastrointestinal diseases, inflammatory diseases, etc.) are expected to have a lower BMI and a higher risk of death [17]. Unfortunately, we do not have data on physical activity, so we could not assess the possible influence of muscle mass and cardiorespiratory fitness in these particularly vulnerable patients. Lower BMI values have been associated with lower muscle mass, leading to reduced mobility and impaired physical performance, conditions associated with a poorer outcome [18,39]. These characteristics may further explain why patients with lower BMI values have a higher risk of dying in the long term after STEMI.

In accordance with previous observations, we found that AKI was associated with worse outcome irrespective of the pathophysiological cause in these patients [1,5,11,32]. Patients with AKI had more than a 2–5-fold higher multivariable-adjusted risk of 30-day mortality and more than a 1.5-fold higher risk of dying in the long term compared to patients without AKI [11]. In addition, we found AKI to be deleterious in all BMI categories, which has also been previously observed in different subsets of patients [21]. Contrary to the analysis by El-Ahmadi et al., we demonstrated that cardiogenic shock was associated with AKI [11]. However, different definitions of AKI and different variables were used in the multivariable model [11]. These differences could explain the diverse results. Other risk factors (age, access site, diabetes, hypertension, hyperlipidemia, and bleeding) have been previously proven to be associated with AKI [6,11]. In addition, as mentioned above, we could not demonstrate any association between BMI categories and AKI.

The result of our study may have potential clinical implications. Our results suggest that STEMI patients with a higher BMI who experience AKI have better survival. Although suggested in guidelines [12], purposeful weight loss without concomitant exercise in these patients may not be beneficial and could even be harmful [16,38,40,41,42,43]. Our study represents a real-world population of patients, including patients with cardiogenic shock and mechanical ventilation, who are mostly excluded from randomized trials. Our finding indicates that the exact mechanism underlying the association between BMI and AKI remains unclear and controversial. The interplay between BMI, AKI, and the risk of death after STEMI needs further investigation to clarify whether this association can be modified therapeutically. Recent data on SGLT2 antagonists and AKI raises hope that we may be able to actively modify AKI in the future [44,45]. Finally, our results suggest that obesity has positive prognostic implications for patients with AKI.

## 5. Limitations

This was a retrospective study of a single center. The observational nature of our analysis allows only limited conclusions about causality, and selection bias could not be excluded. Calculations of GFR were based on creatinine at presentation, which may not have been in a steady state and thus may not be a true estimate of patients’ baseline kidney function. We calculated BMI at hospital admission and could not distinguish between peripheral and abdominal obesity. No data were available on possible changes in BMI during the observation period. We lack data on cardiorespiratory fitness and physical activity before/after MI. Our data included only all-cause mortality, and data on complications from PCI were not available for a sufficient number of patients to be included in the analysis. Data on medications (ACE inhibitors, ARB, statins), hyperuricemia, and glucose were not collected. Data on smoking, previous heart failure, previous revascularization, socioeconomic status, Killip class, or blood pressure, known to be strong predictors of mortality, were missing. Only Caucasians were included in the analysis, making the generalizability of our results questionable. Finally, there were no exclusion criteria for concomitant diseases, so this population represents the real experience of high-risk patients undergoing PCI.

## 6. Conclusions

Our data suggest that higher BMI values (adiposity) are particularly important in patients with AKI and have an additional positive modifying effect in terms of survival. However, the occurrence of AKI in STEMI patients does not depend on BMI. This finding underscores the importance of a multidisciplinary team approach to identify vulnerabilities and tailor preventive measures (hydration, avoidance of nephrotoxic drugs, and prevention of bleeding) and treatment pathways to prevent AKI.

## Figures and Tables

**Table 1:** Patient and procedural characteristics.

	Patients without AKI *n* = 3237 (81.4%)	Patients with AKI *n* = 742 (18.6%)	All Patients *n* = 3979 (100.0%)	*p*
Age (years)	63.2 (12.3)	65.7 (12.5)	63.5 (12.5%)	<0.0001
Male gender	2272 (70.2%)	521 (70.2%)	2793 (70.2%)	1.00
Diabetes	653 (20.2%)	207 (27.9%)	860 (21.6%)	<0.0001
Hypertension	1782 (55.1%)	357 (48.1%)	2139 (53.8%)	0.001
Hyperlipidemia	165 (55.1%)	234 (31.5%)	1889 (47.5%)	<0.0001
CKD	37 (1.1%)	36 (4.9%)	73 (1.8%)	<0.0001
Previous MI	185 (5.7%)	46 (6.2%)	231 (5.8%)	0.60
BMI (kg/m^2^)	27.7 (25.1, 30.7)	27.6 (24.8, 30.9)	27.7 (25.0, 30.8)	0.70
BMI categories				0.13
Normal weight (<25.0 kg/m^2^)	799 (24.7%)	202 (27.2%)	1001 (25.2%)	
Overweight (25.0–29.9 kg/m^2^)	1500 (46.3%)	314 (42.3%)	1814 (45.6%)	
Obesity (≥30 kg/m^2^)	938 (29.0%)	226 (30.5%)	1164 (29.3%)	
Cardiogenic shock	139 (4.3%)	114 (15.4%)	253 (6.4%)	<0.0001
Mechanical ventilation	141 (4.4%)	104 (14.0%)	245 (6.2%)	<0.0001
Creatinine (mg/mL)	0.90 (0.77, 1.10)	0.94 (0.77, 1.21)	0.90 (0.77, 1.12)	<0.0001
GFR (mL/min/1.73 m^2^)	84.4 (65.6, 102.0)	80.7 (55.8, 101.2)	83.8 (64.4, 101.9)	<0.0001
CRP (mg/L)	5.0 (2.0. 15.0)	7.0 (2.0, 24.0)	6.0 (2.0, 16.0)	<0.0001
Radial access	633 (19.6%)	260 (35.0%)	893 (22.4%)	<0.0001
Contrast volume (mL)	150.0 (110.0, 200.0)	158.0 (114.0, 210.0)	150 (110, 200)	0.004
PCI LMCA	66 (2.0%)	28 (3.8%)	94 (2.4%)	0.007
PCI LAD	1378 (42.6%)	340 (45.8%)	1718 (43.2%)	0.11
PCI CX	639 (19.8%)	176 (23.7%)	815 (20.5%)	0.018
PCI RCA	1225 (35.8%)	255 (34.4%)	1480 (37.2%)	0.084
Multivessel PCI	422 (13.4%)	122 (27.5%)	544 (15.1%)	<0.0001
Troponin max (µg/L)	17.7 (5.1, 52.5)	22.7 (6.1, 64.2)	18.9 (5.2, 54.5)	<0.0001
TIMI 0/1 after PCI	140 (4.30%)	41 (5.5%)	181 (4.5%)	0.17
P2Y12	3098 (94.5%)	659 (88.8%)	3717 (93.4%)	<0.0001
EF	47.1 (7.5%)	43.3 (9.6%)	46.4 (8.1%)	<0.0001
Bleeding	223 (6.9%)	159 (21.4%)	382 (9.6%)	<0.0001
Mortality				
30-day death	118 (3.6%)	137 (18.5%)	255 (6.4%)	<0.0001
Long-term death	767 (23.7%)	283 (38.1%)	1050 (26.4%)	<0.0001

Data are expressed as mean ± SD, as a number (percentage), or as the median (interquartile range). AKI = acute kidney injury; BMI = body mass index; CKD = chronic kidney disease; CRP = C-reactive protein; EF = ejection fraction; GFR = glomerular filtration rate; LAD = left anterior descending artery; LMCA = left main coronary artery; P2Y12 = P2Y12 receptor inhibitors; PCI = percutaneous coronary intervention; RCA = right coronary artery; TIMI = thrombolysis in myocardial infarction.

**Table 2:** Independent predictors of AKI in STEMI patients.

STEMI Patients *		
	OR (CI)	*p*
Female sex	0.89 (0.72–1.08)	0.24
Radial access	0.35 (0.25–0.43)	<0.0001
Diabetes	1.36 (1.10–1.68)	0.004
Hypertension	0.72 (0.60–0.87)	0.001
Hyperlipidemia	0.57 (0.47–0.69)	<0.0001
Bleeding	2.51 (1.87–3.68)	<0.0001
Troponin	1.002 (0.999–1.004)	0.13
TIMI 0/1	0.95 (0.64–1.42)	0.82
Contrast volume (mL)	1.001 (1.000–1.002)	0.21
Cardiogenic shock	1.78 (1.26–2.51)	0.001
Mechanical ventilation	1.73 (1.22–2.47)	0.002
CRP	1.002(1.000–1.004)	0.064
Age	1.016 (1.007–1.024)	<0.0001
P2Y12	0.75 (0.54–1.04)	0.087
GFR	1.000 (0.096–1.003)	0.585
BMI category		0.089
Overweight	0.95 (0.76–1.18)	0.63
Obesity	1.20 (0.93–1.54)	0.15

* Normal weight category as a reference. BMI = body mass index; CI = confidence interval; CRP = C-reactive protein; GFR = glomerular filtration rate; OR = odds ratio; P2Y12 = P2Y12 receptor antagonist; STEMI = ST-elevation myocardial infarction; TIMI = thrombolysis in myocardial infarction. Model adjusted for age, sex, hypertension, diabetes, hyperlipidemia, glomerular filtration rate, cardiogenic shock on admission, mechanical ventilation on admission, C-reactive protein on admission, access site, the volume of contrast infused, TIMI 0/1 after PCI, bleeding, troponin peak, P2Y12 receptor antagonists, and BMI classes.

**Table 3:** Associations with 30-day and long-term mortality.

	30-DAY MORTALITY *		LONG-TERM MORTALITY *	
Variable	OR (CI)	*p*	HR (CI)	*p*
Age, years	1.046 (1.029–1.063)	<0.0001	1.57 (1.050–1.064)	<0.0001
Female sex	1.23 (0.86–1.75)	0.25	0.84 (0.73–0.97)	0.019
Diabetes	1.14 (0.78–1.66)	0.49	1.43 (1.24–1.66)	<0.0001
Hypertension	0.72 (0.51–1.02)	0.064	0.90 (0.78–1.04)	0.15
Hyperlipidemia	0.41 (0.27–0.63)	<0.0001	0.70 (0.61–0.82)	<0.0001
BMI		0.26		<0.0001
Overweight	0.76 (0.52–1.12)	0.17	0.73 (0.62–0.85)	<0.0001
Obesity	0.70 (0.44–1.12)	0.14	0.74 (0.62–0.89)	0.002
CRP	1.008 (1.005–1.001)	<0.0001	1.004 (1.003–1.005)	<0.0001
GFR, (mL/min/1.73 m^2^)	0.091 (0.085–0.097)	0.005	0.990 (0.988–0.993)	<0.0001
Cardiogenic shock	4.30 (2.79–6.63)	<0.0001	1.65 (1.32–2.06)	<0.0001
Mechanical ventilation	3.42 (2.15–5.43)	<0.0001	1.66 (1.32–2.09)	<0.0001
Radial access	0.74 (0.48–1.15)	0.18	0.61 (0.50–0.75)	<0.0001
Contrast volume (ml)	1.002 (1.001–1.004)	0.13	1.000 (1.000–1.001)	0.29
TIMI 0/1 after PCI	1.54 (0.84–2.83)	0.116	1.53 (1.21–1.94)	<0.0001
Troponin	1.007 (1.003–1.011)	0.001	1.003 (1.001–1.005)	0.002
P2Y12	0.18 (0.11–0.27)	<0.0001	0.53 (0.43–0.64)	<0.0001
Bleeding	1.55 (1.03–2.35)	0.036	1.79 (1.48–2.16)	<0.0001
AKI	2.59 (1.84–3.64)	<0.0001	1.52 (1.30–1.78)	<0.0001

* Normal weight category as a reference. AKI = acute kidney injury; BMI = body mass index; CI = confidence interval; CRP = C-reactive protein; GFR = glomerular filtration rate; HR = hazard ratio; OR = odds ratio; P2Y12 = P2Y12 receptor antagonist; TIMI = thrombolysis in myocardial infarction. Model adjusted for age, sex, hypertension, diabetes, hyperlipidemia, glomerular filtration rate, cardiogenic shock on admission, mechanical ventilation on admission, C-reactive protein on admission, access site, the volume of contrast infused, TIMI 0/1 after PCI, bleeding, troponin peak, P2Y12 receptor antagonists, BMI classes, and AKI.

**Table 4:** Association between BMI and 30-day and long-term mortality in STEMI patients with AKI.

	STEMI patients with AKI			
	30-DAY MORTALITY *		LONG-TERM MORTALITY *	
Variable	OR (CI)	*p*	HR (CI)	*p*
BMI		0.20		0.022
Overweight	0.85 (0.46–1.58)	0.61	0.76 (0.57–1.02)	0.067
Obesity	0.51 (0.23–1.09)	0.082	0.62 (0.44–0.88)	0.007

* Normal weight category as a reference. AKI = acute kidney injury; BMI = body mass index; CI = confidence interval; HR = hazard ratio; OR = odds ratio; STEMI= ST-elevation myocardial infarction. Model adjusted for age, sex, hypertension, diabetes, hyperlipidemia, glomerular filtration rate, cardiogenic shock on admission, mechanical ventilation on admission, C-reactive protein on admission, access site, the volume of contrast infused, TIMI 0/1 after PCI, bleeding, troponin peak, P2Y12 receptor antagonists, and BMI classes.

## Data Availability

All data are available from the corresponding author upon reasonable request.

## Supplementary Materials

The following supporting information can be downloaded at: https://www.mdpi.com/article/10.3390/jcm12237311/s1, Table S1: Patient and procedural characteristics in different BMI categories.
