# Supplementary material for: Obesity and Acute Kidney Injury in Patients with ST-Elevation Myocardial Infarction
Source: J Clin Med. 2023 Nov 25;12(23):7311. doi: 10.3390/jcm12237311 (PMC10707242; doi:10.3390/jcm12237311)
Supplement: Supplementary file 1 [file jcm-12-07311-s001.zip › jcm-2677594-supplementary.pdf]

Table S1. Patient and procedural characteristics in different BMI categories.

	**Normal-weight patients (BMI < 25.0 kg/m²) N= 1001**	**Overweight patients (BMI = 25.0–29.9 kg/m²) N = 1814**	**Patients with obesity (BMI ≥ 30 kg/m²) N= 1164**	**All patients N= 3979**	**p**
Age (years)	66.0 (13.2)	63.4 (12.4)	61.6 (11.6)	63.5 (12.5)	<0.0001
Male gender	626 (62.6%)	1347 (74.3%)	820 (70.4%)	2793 (70.2%)	<0.0001
Diabetes	128 (12.8%)	373 (20.6%)	359 (30.8%)	860 (21.6%)	<0.0001
Hypertension	428 (42.8%)	958 (52.8%)	753 (64.7%)	2139 (53.8%)	<0.0001
Hyperlipidemia	421 (42.1%)	878 (48.4%)	590 (50.7%)	1889 (47.5%)	<0.0001
CKD	26 (2.6%)	28 (1.5%)	19 (1.5%)	73 (1.8%)	0.11
Previous MI	55 (5.5%)	110 (6.1%)	66 (5.7%)	231 (5.8%)	0.80
BMI (kg/m²)	23.6 (22.1, 24.4)	27.5 (26.2, 28.7)	32.7 (31.2, 34.9)	27.7 (25.0, 30.8)	<0.0001
Cardiogenic shock	69 (6.9%)	121 (6.7%)	63 (5.4%)	253 (6.4%)	0.28
Mechanical ventilation	73 (7.3%)	113 (6.2%)	59 (5.1%)	245 (6.2%)	0.098
Creatinine (mg/mL)	0.87 (0.72, 1.09)	0.99 (0.82, 1.28)	0.93 (0.78, 1.12)	0.90 (0.77, 1.12)	<0.0001
Total cholesterol (mmol/L)	4.72 (3.80, 5.60)	4.732 (3.90, 5.60)	4.80 (4.01, 5.68)	4.75 (3.90, 5.61)	0.037
Triglycerides (mmol/L)	1.00 (0.70, 1.60)	1.25 (0.80, 1.82)	1.40 (1.00, 2.30)	1.22 (0.81, 1.90)	<0.0001
HDL-cholesterol (mmol/L)	1.12 (0.90, 1.36)	1.03 (0.86, 1.23)	0.99 (0.82, 1.20)	1.04 (0.86, 1.25)	<0.0001
LDL-cholesterol (mmol/L)	3.10 (2.30, 3.80)	3.14 (2.40, 3.90)	3.10 (2.47, 3.80)	3.10 (2.40, 3.80)	0.28
GFR (ml/min/1.73 m²)	84.9 (62.4, 105.7)	83.6 (65.5, 100.3)	83.8 (63.7 101.0),	83.8 (64.4, 101.9)	0.26
CRP (mg/L)	5.0 (2.0,16.0)	6.0 (2.0,18.0)	6.0 (2.0,16.0)	6.0 (2.0, 16.0)	0.009
Radial access	200 (20.0%)	400 (22.1%)	293 (25.2%)	893 (22.4%)	0.013
Contrast volume (ml)	144.0 (110.0, 193.0)	150.0 (110.0, 200.0)	158.0 (115.0, 210.0)	150.0 (110.0, 200.0)	<0.0001
PCI LMCA	32 (3.2%)	42 (2.3%)	20 (1.7%)	94 (2.4%)	0.08
PCI LAD	446 (44.6%)	777 (42.8%)	495 (42.5%)	1718 (43.2%)	0.59
PCI CX	185 (18.5%)	384 (21.2%)	246 (21.1%)	815 (20.5%)	0.19
PCI RCA	359 (35.9%)	690 (38.0%)	430 (37.0%)	1480 (37.2%)	0.52
Multivessel PCI	134 (14.7%)	258 (15.6%)	152 (14.8%)	544 (5.1%)	0.80
Troponin max (µg/L)	21.1 (5.1, 55.7)	18.5 (5.4, 56.4)	17.5 (5.2, 50.4)	18.9 (5.2, 54.5)	0.19
TIMI 0/1 after PCI	41 (4.1%)	84 (4.6%)	56 (4.8%)	181 (4.5%)	0.71
P2Y12	935 (93.4%)	1690 (93.2%)	1092 (93.8%)	3717 (93.4%)	0.78
EF	45.8 (8.6%)	46.4 (8.1%)	46.9 (7.5%)	46.4 (8.1%)	0.01
Bleeding	128 (12.8%)	171 (9.4%)	83 (7.1%)	382 (9.6%)	<0.0001
Mortality outcome					
30-day death	84 (8.4%)	112 (6.2%)	59 (5.1%)	255 (6.4%)	0.005
Long-term death	344 (34.4%)	445 (24.5%)	261 (22.4%)	1050 (26.4%)	<0.0001

Data are expressed as mean ± SD, as a number (percentage), or as the median (interquartile range). BMI = body mass index; CKD = chronic kidney disease; CRP = C-reactive protein; EF = ejection fraction; GFR = glomerular filtration rate; LAD = left anterior descending artery; LCX = circumflex artery; LMCA= left main coronary artery; P2Y12 = P2Y12 receptor inhibitors; PCI = percutaneous coronary intervention; RCA = right coronary artery; TIMI = Thrombolysis In Myocardial infarction.
